# The process of care in integrative health care settings – a qualitative study of US practices

**DOI:** 10.1186/1472-6882-14-410

**Published:** 2014-10-23

**Authors:** Suzanne J Grant, Alan Bensoussan

**Affiliations:** National Institute of Complementary Medicine, University of Western Sydney, Locked Bag 1797, Penrith, NSW 2751 Australia

**Keywords:** Integrative healthcare, Integrated healthcare, Complementary and alternative medicine

## Abstract

**Background:**

There is a lack of research on the organisational operations of integrative healthcare (IHC) practices. IHC is a therapeutic strategy integrating conventional and complementary medicine in a shared context to administer individualized treatment. To better understand the process of care in IHC - the way in which patients are triaged and treatment plans are constructed, interviews were conducted with integrative health care leaders and practitioners in the US.

**Methods:**

Semi-structured interviews were conducted with a pragmatic group of fourteen leaders and practitioners from nine different IHC settings. All interviews were conducted face-to-face with the exception of one phone interview. Questions focussed on understanding the “process of care” in an integrative healthcare setting. Deductive categories were formed from the aims of the study, focusing on: organisational structure, processes of care (subcategories: patient intake, treatment and charting, use of guidelines or protocols), prevalent diseases or conditions treated, and the role of research in the organisation. The similarities and differences of the ITH entities emerged from this process.

**Results:**

On an organisational level, conventional and CM services and therapies were co-located in all nine settings. For patients, this means there is more opportunity for ‘seamless care’. Shared information systems enabled easy communication using internal messaging or email systems, and shared patient intake information. But beyond this infrastructure alignment for integrative health care was less supported. There were no use of protocols or guidelines within any centre, no patient monitoring mechanism beyond that which occurred within one-on-one appointments. Joint planning for a patient treatment was typically ad hoc through informal mechanisms. Additional duties typically come at a direct financial cost to fee-for-service practitioners. In contrast, service delivery and the process of care within hospital inpatient services followed a more formalised structure.

**Conclusions:**

IHC is a complex, emerging field with divergent meanings and interpretations. The structures and processes of the IHC entities reported provide insight to the variable ways in which IHC manifests whilst commonly holding a similar vision. This report contributes to understanding IHC, providing evidence for future planning, implementation and evaluation to meet patient needs and demands in this area.

## Background

Integrative healthcare aims to treat the whole person (physical, emotional, energetic, spiritual), using the body’s innate ability to heal itself with a blend of conventional and complementary therapies [1, 2]. This style of healthcare brings together different health professionals with differing levels of expertise. Integrating the different expertise and “unique insights” of this divergent group to form a comprehensive process of care and treatment plan for patients can be challenging [1, 3]. This paper reports on the process of care used in key integrative medicine facilities in the US.

There is an overall lack of research on the organisational operations of integrative healthcare (IHC) practices [4, 5]. To better understand the process of care in IHC - the way in which patients are triaged and treatment plans are constructed - interviews were conducted with integrative health care leaders and practitioners in the US. The focus was on integrative health care defined as a patient-centred, inter-disciplinary approach where there is a combination of conventional medicine with complementary and alternative medicine, and co-management is desired or occurring at some level. This circumstance allows each provider and each patient to contribute their knowledge, particular skills and preferences to focus on providing health care to persons within individual care plans. As an emerging field, attempts to implement IHC have been made [6–8] but reviews or summaries of this field are sparse, and none have focused specifically on the process of care [9]. We need to understand the process of care in IHC that people have adopted to understand which aspects are the most or least successful. In this way we may be able to trial IHC as a therapeutic strategy, ascertain safety, efficacy and cost effectiveness for chronic and complex conditions.

## Methods

The study sample was a pragmatic group selected from integrative medicine centres and integrative healthcare leaders in the US. We sought to cover a range of centres, those which were primary care only, those with consultant care only, those attached to Universities or hospitals. Fourteen IHC leaders in nine University-based clinics, privately owned primary care clinics and hospital based clinics were approached and all agreed to be interviewed. These centres were identified through discussions with integrative Medicine (IM) leaders and were geographically convenient. Two executive directors of IM centres, eight integrative medicine physicians, and four complementary medicine practitioners were interviewed. Written informed consent was provided by all of the interview partners. All interviews were conducted face-to-face with the exception of one phone interview. A summary of the centres represented is provided in Table 1.

**Table 1:** **Key characteristics of care of IHC centres**

Centre	Business structure	Practitioners and services	Summary of clinical process	Research performed
UCLA Centre for East–west Medicine	Consultant care, University based P/T and F/T salaried CM staff	Practitioners: 6 MDs (salaried), 5 clinical specialists (salaried acupuncturists, &/or massage therapists)	Patients may be referred to the clinic or attend directly. There are around 500 physicians who refer directly to the Centre. An initial patient intake form is provided and the patient seen by an MD with some background in Chinese medicine. Treatment is individualised and provided by the dual trained physician or through referral from the MD to one of the other practitioners. No protocols or guidelines are used.	Yes
		Services: comprehensive consultation, patient education on self-care techniques, medication adjustments, lifestyle coaching and diet recommendations, acupuncture, trigger point injections, and myofascial release/therapeutic massage. The Centre is in the process of being able to offer primary care services in addition to consultative.	The full implementation of Electronic Health Records (EHR) occurs late 2013. Currently, patient charts are on paper but will transition into electronic copies. When EHR is implemented, charting, scheduling, notes, pharmacy requests, and et cetera will all be completed electronically. Every patient encounter will be logged, monitored, and shared with the next practitioner at UCLA Health.	
		Special clinics: Breast Cancer, Head and Neck diseases, Inflammatory conditions, Oesophageal disorders		
The Penny George institute for Health and Healing - Abbott Northwestern Hospital	Hospital inpatient	The hospital has an integrative medicine team of 7 experienced acupuncturists and other CM practitioners including aromatherapy, art & music therapy, guided imagery, energy healing, massage, Korean hand therapy, reflexology and relaxation techniques. Staff are salaried.	Hospital: Referrals are provided by nurse or physician, the patient may also request the service but it must have any treatment from the acupuncturist signed off by the physician. Some guidelines for referrals for pain, anxiety, elimination problems, nausea or vomiting. Common charts are used which record time, reason, SOAP, pain, anxiety and nausea scores. Morning meetings between the IM team enable patients to be discussed and allocated. Biweekly case conferences. Practitioners have training in IM principles and modalities and team work. CM practitioners may ‘round’ with conventional medical practitioners. Systematic recording of before and after of pain.	Yes
	Part of Allina Health			
	Salaried CM staff.			
	Outpatient clinic Part of Allina Health	The Outpatient Clinics offer individual and group acupuncture sessions, Traditional Chinese Medicine, resilience training, nutritional counselling, Mindful eating workshops, massage, wellness visits, exercise physiology consults, biofeedback	Clinic: referrals or self-referral. Patients may have a joint consult with an MD and nurse practitioner or clinician to understand integrative health therapies.	
	Salaried or Fee for service?			
Beth Israel Hospital	Hospital Part of Continuum Health Partners Inc (not for profit org) Volunteer and paid CM staff	Practitioners: Acupuncturists, Reiki practitioner, Yoga therapists	Inpatient services can be accessed by contacting the program coordinator or by referral from other staff. Acupuncture services require sign off by the physician. The hospital operates a volunteer acupuncturist intern program at the hospital. The acupuncturists treat pain, anxiety and nausea. Systematic recording of before and after of pain. Details, history and documentation of the program are shared.	Yes
		Services: Holistic Preparation for Surgery Program, Acupuncture, yoga therapy program, healing touch program, contemplative care services		
	Primary care consultants P/T and F/T CM staff	Practitioners: IM MDs, MDs with speciality areas including cardiology, ENT, sports medicine and rehabilitation, Gynaecologist, Podiatrist, Physical Therapist, Nutritionist, Chiropractor (also an acupuncturist), Acupuncturists, Nurse Practitioners (one is also a homeopath) ,	Patients can make an appointment with any practitioner, be referred internally or externally. Practitioners share common electronic medical records. Practitioners have own offices and treatment rooms. Fortnightly meetings are held for practitioners and others to attend. Topics may be an integrative approach to a particular condition or complex cases may be presented for feedback.	Yes
		Services: internal medicine, cardiology, dermatology, family medicine, orthopaedic and sports rehabilitation, physical Therapy, occupational and hand therapy, gynaecology, ear, nose and throat; chiropractic; podiatry, nutrition, psychotherapy and stress management, acupuncture and East Asian medicine, massage, integrative therapies, fitness centre, yoga therapy, mindfulness meditation, Dean Ornish Heart Disease Reversal Program, fitness centre		
Casey Health Institute	Family medical centre, privately owned P/T and F/T salaried CM staff	Practitioners: MDs, Nurse practitioner, Naturopath, Chiropractor, Acupuncturist, Massage therapist, Reiki master, Nutritionist, Psychologist,	Patients can make an initial appointment with any practitioner or schedule wellness service/class. There is no physician gatekeeper. Practice is organized around a central team room where practitioners are constantly discussing integrative treatment of patients informally and cross referring In addition to regular weekly required Integrative Case conferences and separate weekly integrative didactic meetings. Shared electronic medical records with capability of tracking medical and economic outcomes data. All practitioners have access to records. One of a small number of Integrative Primary Care centres that takes all insurance, Medicare, Medicaid and will also be a level III Patient Centered Medical Home.	Yes
		Services: primary care, acupuncture, massage, naturopathy, chiropractic, psychology, therapeutic yoga, fitness classes, health coaching, patient navigator, pilates, zumba, reiki, a range of wellness workshops		
		Staff are salaried, mostly full time.		
IMCSR	Family medical centre, privately owned P/T and F/T staff are fee for service	Integrative medicine physician, two naturopaths, acupuncturist, a feldenkrais practitioner, nutritionist and chiropractor. Staff are fee for service.	Self-referral to any practitioner. IM physician may suggest treatment with other practitioners. Communication about patients through shared medical records? Offers a free-of-charge 10–15 min consultation to clarify which provider and/or service. Another option is to use a patient navigator (licensed social worker) who is available, for a fee, to offer guidance about choices of modalities, practitioners and approaches to your health and healing. There are some individualised integrative programs Pain for a fee of $4000-$6000. Patient Care Conferences are available where all practitioners and the patient meet for a fee of $650-, there is some rebate from insurance but minimal. The demand for these services is not so high due to the cost.	No
		Services: primary care, acupuncture, naturopathy, chiropractic, feldenkrais, and a range of special programs are run from time to time such Pain Rehabilitation, Mindfulness-based stress reduction, Sleep Health.		
Osher Centre	Consultative integrative care University affiliated (UCSF)	Physicians: 2 IM family physicians, 2 integrative oncologists, 1 manual medicine physician, 2 integrative paediatricians, 1 integrative psychiatrist, one women’s health naturopath	The Osher centre runs an integrative clinic on a consultation basis. Integration is informal and physician led. The clinic is currently in the process of moving to electronic medical records (EMR) and it is thought this will make integration easier with shared charts. The care of a patient with a chronic condition might be led by either a physician or non-physician. The lead physician documents a treatment plan that might involve going to other practitioners for treatment and returning for a review in a set period of time. The practitioner works out a specific treatment plan but does not always communicate this back to the lead physician except perhaps incidentally. Patient review and progress is monitored by the lead physician. Case conferences are mandatory but only a limited number of cases are presented.	Yes
	CM staff are P/T & F/T salaried	Non-physicians: 2–3 acupuncturists, 2 massage therapist, 1 biofeedback therapist, 1 integrative psychotherapist		
		Services and classes: guided imagery, laughter yoga classes, tai chi, yoga		
Scripps Centre for Integrative Medicine	Consultant care, Part of Scripps Health (not for profit); P/T & F/T CM staff are fee for service	Practitioners: IM physicians in cardiac care, pain, and headache. Biofeedback practitioner, massage therapist, dietician	The Scripps Centre for Integrative Medicine runs on a consultation basis. Referral is by self or specialist. Lead practitioner is typically an MD. A treatment plan may involve a dietician’s consultation, supplements (prescribed by MD), a mind-body method and perhaps biofeedback sessions.	Yes, some.
		Services and classes: consultant care, acupuncture, medical hypnosis, biofeedback, massage, stress reduction programs for pain or other problems, lifestyle change program, fitness centre.		
Susan Samueli Centre (California)	Consultant care, University affiliated (UCI School of Medicine) P/T & F/T CM staff are fee for service	An IM physician, naturopath, acupuncturist and massage therapist.	IM physician oversees and directs treatment, integration is informal. The overall treatment plan is coordinated with each involved practitioner and treatment is constructed according to the individual presentation and patient preference. Shared paper based records. Providers are paid based on hours spent in the clinic seeing patients.	Some
University of Maryland Centre for Integrative Medicine	Consultant care University based; P/T & F/T CM staff are salaried	Practitioners: IM physicians, Osteopaths, Acupuncturists, Chinese medicine practitioners, Psychologist, Qi Gong and Tai Chi instructors, Reflexologist and Reiki practitioner, massage therapist.	Referrals from other practitioners or self-referral to any practitioner. Initially ran integrative programs, now operates with an IHC team but joint meetings are infrequent and shared case management not common. The Clinic is in the process of moving toward shared electronic health records. Consultants work within the clinic. Providers are paid based on hours spent in the clinic seeing patients. Consultation/Collaboration?	Yes
		Services: consultant care, acupuncture, psychotherapy, homeopathy, reflexology, life coaching, qi gong, tai chi, nutrition.		

An interview guideline was developed based on a review of literature and discussions with key people within integrative health care. All interviews were conducted by one interviewer (SG) from May to June 2012. Interviews lasted for 1–2 hours. Questions focused on gaining a brief understanding of the operational structure of the clinic but more on the processes of care: How are patients managed within an integrative setting? Who is responsible for the treatment plan? How is this treatment plan constructed? Are they undertaking any research on integrative approaches? With a view to “testing” an integrative health care approach in a research setting, discussions also focused on conditions that were most suited to an integrative approach.

All interviews were recorded. Interviews were transcribed verbatim by an external party. The analytical approach was deductive. We used the framework established from the study aims and the questions which had been constructed to achieve these aims [10, 11]. Categories for indexing data were formed at the beginning: organisational structure, processes of care (subcategories: patient intake, treatment and charting, use of guidelines or protocols), prevalent diseases or conditions treated, and the role of research in the organisation. The themes associated with these categories were coded. Coded data from the transcriptions was transferred into the categories within an electronic table format, similar to Table 1 but with additional headings for the different categories. Coding and indexing was carried out by one researcher (SG) and reviewed by the other (AB). We anticipated that the IHC entities would have overlapping but distinct characteristics.

## Results

All nine IHC clinics and hospitals were selected and based in the US. Results are summarised by way of structure, process of care, prevalent diseases or conditions treated and research.

### Organisational structure

Of the nine IHC entities, two operated hospital inpatient IHC and clinics or centres external to the hospital. Four IHC centres were based in a University or within a University network. Two were part of a large private health organisation and two were independent private clinics (Table 1).

All consultant care and family medicine centres had CM practitioners co-located with biomedical practitioners within the same building using a single reception. Practitioners used individual treatment rooms, and most practitioners completed charts in this same room with the exception of two centres. One of these provided an office in addition to treatment space, and the other sought to facilitate sharing by providing a common room for all practitioners (both CM and biomedical) to chart.

In hospital based integrative care, CM practitioners were mobile depending on demand. CM practitioners were typically either part-time or full-time employees of the hospital or affiliated University. In one case, the IM practitioners were part of an unpaid volunteer program. CM practitioners chart at stations around the hospital. Both hospital IM services were overseen by a specific coordinator. One hospital allocated new referrals to an IM practitioner who served as their care coordinator during their hospital stay.

In consultant care and family medicine clinics, CM practitioners worked part-time as either contractors (fee-for-service), running their own private practices within the clinic or as salaried employees.

“Everybody who is in this clinic is part time. We don’t have anybody who is full time except for our front office person. I’m here only 60% of the time, my naturopath is 70% time, my acupuncturist is 40-50% time and my massage service is 30% time. …They are not university employees. They get paid according to the patient load and according to the hours that they spent here depending on how the contract was drawn. For us, for now, it’s a good model because having somebody as an employee means one: that you are committed to their entire salary, plus benefits, plus everything else that you have to do, which basically means that you’re sort of doubling the amount that you’re paying them and that, we can’t afford. For us, for the time being this is a viable model”. (Int1)

CM practitioners typically had private clinical practices outside of the clinic or hospital. Philosophically, IHC clinics had similar visions. The approach was about supporting patients holistically to ‘restore health’ or achieve a ‘balance of mind, body and soul’. These centres sought to achieve this through the provision of a wide array therapies and practices to ‘optimise health’ , and meet patient needs. All practitioners and leaders in IM agreed that the mind-body modalities are important in encouraging behaviour change and developing resilience. Often reference was made to helping patients’ to “find meaning and purpose”, “facilitating self-care”, “harnessing salutogenesis” (Int6).

#### Types of CM practitioners

Family medicine and consultant care clinics varied in the composition of CM practitioners. All centres except one had at least one massage therapist, one acupuncturist or East Asian medicine practitioner, and the majority had a naturopath. Other services offered included reiki, reflexology, nutrition, and biofeedback. Many centres also offered mind-body workshops, yoga, qigong or tai chi.

### Process of care

#### Patient intake or triage

Referrals to IHC clinics come from other physicians, specialists or from the patient themselves. One clinic operates as a specialist centre:

“…referrals come from primary care physicians or neurologists, usually when there is depression, or the patient doesn’t want to continue to take pharmaceuticals or there is stress. Or when the typical pain preventers are not working. The patient’s (electronic) medical record is shared so all the IM practitioners …are familiar with what they are taking”. (Int5)

Within the hospital settings, referral to an IM service was done by a nurse or physician. Patients could also request an IM service. The referral might have a basic explanation; the rest is learned from the patient chart or nurse on the floor. Patients have to sign written consent for acupuncture but not for any other IM.

Within consultant care and family medicine clinic, intake usually involved a holistic questionnaire followed by a long consult. An examination of intake forms revealed similar intent to capture diet, lifestyle, and history in detail. In some cases the clinic posted out the intake forms prior to the first appointment to ensure there was plenty of time to complete the form prior to the first consult. Initial consults typically lasted from 20 minutes to an hour with one practitioner only.

In most cases when new patients arrived at an IHC primary care centre via a referral from another physician, or specialist, the patient was seen by a biomedical practitioner (an MD) first. In other instances where contact was initiated by the patient, the patient nominated a practitioner they wished to see. In some cases, reception staff directed the patient to a physician or practitioner based on availability and suitability. It was more common to see the physician first, who would then “broker” their treatment. Referrals from specialists and physicians to the CM practitioner from outside the clinic were not common.

One family medicine clinic had pioneered a novel approach where the patient was initially seen by a “patient navigator”. Initially the patient navigator was part of the IM centre but this was too costly. Now the patient may pay a fee to have a consultation with a navigator to assist in guiding care. This approach was raised as desirable, at least in theory, by other leaders and practitioners in the IM field. Ideally a “patient navigator”,

“…would be somebody inside a clinic who does that, so it’s your integrative health care coach, or your integrative health care manager. It’s not a physician. I think that’s too costly …a nurse could probably do this. They have the clinical expertise, they have the orientation frequently of being person-centred and trying to listen to those areas and they also need to be empowered in this area and would probably love to be empowered in this area. But, they would have to be trained to apply a triage system and how to customize that to the patient and then to track the patient …they’re integrating it so it remains patient-centred and it works with the patient to decide what’s working with what patient within the team….Everybody’s competing for that position because one of the things the Health Reform Act is probably going to require is someone who’s the system integrator, patient advocate”. (Int10)

There was a general consensus that the skills and knowledge required to triage a patient and develop an IHC treatment plan are fairly broad but a nurse practitioner could be trained to provide this service. One IM leader suggested that those who are experts within the IHC for the main presenting condition should do the triage and treatment plan in consultation with others; for example, a naturopath may be an appropriate practitioner to triage those with IBS. The extent to which an “integrative” approach was necessary was thought to vary depending on the presentation of the patient:

“So, I don’t know why an experienced Nurse Practitioner (NP) couldn’t offer [triage]…then does the MD just come in for when things aren’t going right, or it got complicated? Or, does the MD maybe serve people with more co-morbidities, or is there a triage function where, you know if the NP does the evaluation and there is a lot of complex co-morbidities and medications then one of the first steps is going to the MD. Whereas, if it’s a straightforward case of back pain with minimal co-morbidities and not a lot of medications, then you know, less integrative care is needed”. (Int4)

#### Treatment and “charting”

In primary care IHC settings, the treatment plan was largely overseen by the biomedical physician.

“Every patient that we see, when they walk out of the clinic, they walk out with something that we call an “Action Plan”. Which basically summarizes all the medications that they are currently taking, all the supplements they are currently taking, any modifications that I need to do, how they are supposed to take it and then any labs, dietary changes, lifestyle changes and when they are supposed to follow up, and who they are to see. Everybody walks out with an “Action Plan” every time they see us. …” (Int1)

All the leaders and IM practitioners interviewed approached treatment planning with the patient at the centre. Patient preferences for treatment modality are considered along with the social and financial situation of the patient. These preferences were viewed as central the treatment plan. Although no centre had any formal structure in place to facilitate this, rather it was acknowledged as part of the dialogue of the individual consultation.

Functional pathology results were also commonly used to guide the initial treatment plan, although use was dependent on the insurance coverage for these tests. If it was available then the pathology for nutrient deficiencies would be used to individualize supplements.

Charting and patient reviews were not so uniformly conducted. Charting was considered an important way to share information. To this end many IHC centres used the same electronic medical records system for their patients. All EMR systems assisted sharing, some to a greater degree than others. A few practices did not have a shared system and relied on emails for communication between practitioners or paper based systems. Other methods of sharing information about patients included “corridor conversations”, arranging quick catch up meetings or case conferencing or a combination:

“I just bump into them in the hall or we email. Then we have a Case Conference twice a month, where we’re together and it’s an opportunity to speak also. Equally frequently, I make quick meetings with people so, ‘For 15 minutes tomorrow, let’s talk’ ”. (Int2)

Practitioners within IHC centres, constructed and reviewed the treatment plan according to how the patient responds or they might alter their approach based on how another practitioner within the centre assesses the patient. Within hospital based IHC services the treatment plan was targeted to specific symptoms, primarily to reduce pain, anxiety, nausea/vomiting and discomfort. IHC practitioners were able to tailor their treatment according to their skills and experience to meet the needs of the patient. No centre had a protocol in place that facilitated the review of a treatment plan of a patient receiving integrative care.

“Case conferences” were currently being held in all IHC centres or had been held in the past. The conferences served different purposes. The following comment was typical of how case conferences run

…so everybody in the room absolutely…all the practitioners (12 different ones) they all believe they can absolutely help every patient, with small exceptions. So, everybody sees how they can help that patient. It always comes down to what’s that patient’s interest, readiness, past history, money, what’s covered, what isn’t covered, all that kind of thing. Because that determines the first 2 or 3 people that they’re going to see more than anything else. (Int3)

Other practices held intermittent meetings to discuss best evidence or “difficult” cases. There was a sense that these case conferences were quite powerful. One interviewee noted that “It’s amazing the progress that gets made in those sessions…The creativity and the ideas that come up”. Only one centre had a system where, for a fee, the patient may be present at these case conferences.

Many in clinical practice reflected that they didn’t perhaps meet together often enough. In the history of one clinic, meetings were facilitated around one topic such as pain. The “pain” group would meet together to discuss difficult patients. One interviewee noted that these meetings were fruitful but “in hindsight, perhaps the only thing missing was the patient”.

A common theme that emerged was that collaboration was often driven by the type of business model of the centre. Regular meetings were much easier to schedule in centres with salaried practitioners. Practitioners not on salary, typically needed to attend the case conferences on their own time. Most CM practitioners typically working within the IM settings are part-time contractors they do not have the time or resources required to participate in case conferences. There was a preference to have IM practitioners on staff if it was financially viable so as to facilitate integrative processes.

Case management was also influenced by the US regulatory system. Two interviewees noted that it does not favour having a CM practitioner as the “managing” practitioner. Where an individual has private health insurance coverage for a CM therapy this assists in making IHC more financially viable and accessible.

#### The use of guidelines, protocols and programs

Interviewees involved in clinical practice were asked whether they had developed or used fixed programs, guidelines or protocols to help with treatment. Many of the centres visited had “dabbled” in running a set integrative program targeting specific conditions such as “pain” or “weight loss”. Such a program may integrate educational, spiritual and nutrition components alongside other interventions. But,

“people were not willing to spend that much money for a bundled program. Particularly because many of them were not sure if they were going to stick with it or not. (The weight loss program) would consist of meeting with one of the providers on a regular basis…We…offered free measurements during those educational classes for people to become aware of their body fat, water content, but we were not able to sell that idea. Then we tried to scale it down to shorter versions, so instead of involving everybody in the clinic in the program, we tried to scale it down to seeing one or 2 people. It still didn’t work. Again, I’m not sure if it is just the timing because when we started thinking and developing a program like the economy was turning and people were really mindful of how they wanted to spend their money”. (Int1)

Those centres that had run fixed programs or services around a condition were more partial to providing services such as mindfulness programs, yoga classes or tai chi. With these services they could be added onto a patient’s individualised program, rather than trying to ‘squeeze’ the individual into a fixed program.

A few interviewees had experimented with protocols or guidelines. The response to the usefulness of guidelines was mixed.

“The problem with some EBM guidelines is that they are directed to the “simple patient”…after you get to a certain complexity you have so many different potential derivations that it’s impossible”. (Int5)

“We tried this… to develop a sort of a protocol for care and say “For this, we do this”, but, over time we have found out that, each person is very different and we have to be very fluid, and allow for the ability to modify and change the protocol without being locked into one.” (Int1)

Another problem noted with using a protocol or guideline in practice was that treatment choice is ultimately restricted by the ability to pay. The pathway chosen by a patient may not reflect what they want but rather what their insurance covers.

There is considerable effort being made through the BRAVENET initiative, Patients Receiving Integrative Medicine Interventions Registry (PRIMIER), to collect prospective data about IM patients using the Patient-Reported Outcome Measurement System (PROMIS) measures and examining the natural history of IM interventions. Some leaders consider that when enough data is collected a mapping of the treatment and outcomes used for people with a particular condition, a “protocol” for the treatment may emerge and then a trial would be feasible.

IM leaders and practitioners were asked to reflect on the usefulness of ‘process of care’ protocols, an algorithm to guide a patient in an IHC setting. The response was mixed. Interviewees did not see usefulness in a protocol that prescribed the ‘content of care’. Some considered protocols an inadequate reflection of what occurs in real life practice:

“…even if you were to pick 2 protocols for treating neck pain and you felt like you had good evidence and a pile of data…you’re still only looking at 2 protocols for one treatment in this vast world of options. You’re still not taking advantage of this incredible wealth of clinical kind of experience that’s developing…” (Int4)

Labelling patients and using standard biomedical disease names was also considered another obstacle to the usefulness of protocols. Patient presentation may be complex and involve many different factors such as depression, insomnia or when the condition doesn’t fit neatly into one title:

“..like central sensitization syndrome… I don’t know what the right term would be that someone reading your guidelines would recognize. It’s different to chronic pain …” (Int5)

“(It is difficult to) use a specific algorithm, or a specific protocol to follow for a disease name. An individual’s spiritual and emotional work will be different from another’s….Stress…might be one thing for you, it might be completely different for me, it might be completely different for someone else. It might be a combination, even back pain and stuff like that”. (Int7)

Hospital IHC services had guidelines for what conditions were considered suitable for referral to a CM practitioner. Once referred the treatment protocol was individualised.

### Prevalent diseases or conditions treated

Interviewees were asked to comment on conditions they commonly treat in IHC primary care settings. These were chronic conditions such as irritable bowel syndrome, fibromyalgia, chronic fatigue, pain, back pain, obesity and headaches. In considering which conditions may be suitable for investigation in a clinical trial of IHC, conditions where there was a body of evidence for CM were thought to be best. These included headache, back pain, arthritis and diseases across the metabolic spectrum.

There was some consensus that approaching a trial of IHC with the view to treating a single symptom or disease was not appropriate. The strength of IHC is the incorporation of the whole body in diagnosis and treatment. For example it might be best to assess and treat a disease spectrum or the common factor across the diagnoses such as “inflammation” or “stress”.

“..we need to stop talking about PTSD or TBI (traumatic brain injury) although those are important to assess in themselves, but start talking about the trauma response and specifically call it the trauma spectrum response, so that it communicates to folks that there’s a spectrum of co-morbidities that go together…the physiological mechanisms for those co-morbidities have common foundations mechanistically within the body, in terms of stress and stress response… they all run together”. (Int6)

There was strong agreement that treatment in an IHC setting focuses on the whole person and thereby addresses co-morbidities and encourage salutogenesis.

Like, we give magnesium for headaches and their headaches may be not statistically significantly better but that magnesium actually improved their muscle tension or their sleep. It may have a slight anti-depressant effect. (Int5)

By virtue of the fact that the treatments… that many of these treatments, not all of them but many of them are designed to facilitate their own healing process. It’s a key issue, I think. (Int6)

Many leaders and clinics considered a mind-body intervention as a central component to an IHC treatment and should be part of any trial conducted in this field. This part of the intervention could be conducted using an off the shelf course such as Mindfulness Based Stress Reduction (Jon Kabat-Zinn) or a modified version [12]. Reference was also made to a Resilience Training program, an 8 week integrative intervention for people with depression, there is some individualisation and some set components [13]. However, interviewees also noted that a sitting meditation may not suit everyone, others may be better with tai chi or yoga.

Within the two hospital IHC inpatient settings, the respondents indicated that the focus of the CM modalities was not on treating the specific ‘disease’ but mitigating side effects and facilitating comfort. Typically the treatment focus was on pain, nausea/vomiting, anxiety, fatigue and other symptoms.

### Research

All interviewees were asked if they systematically and regularly assessed patient health outcomes in their practice. Five of the nine centres visited are collecting patient outcome data as part the PRIMIER. This is a practice-based research network that is part of BraveNet. The initiative utilises data extracted from electronic medical records and patient-reported outcomes using the Patient-Reported Outcome Measurement System (PROMIS). PROMIS is a web-based survey that collects patient reported data on quality of life, fatigue, pain, depression and physical function. This is matched to data collected on the patient’s medical record such as tests, costs, and other routine measures collected during consultations. BraveNet has also undertaken a specific pain study across several of the partner centres. The Study on Integrative Medicine Treatment Approaches for Pain (SIMTAP) collected information on the integrative treatment of 400 patients with chronic pain. Interviewees supported such networks and considered them a feasible way of providing some evidence about integrative health care outcomes.

Interviewees not involved in PRIMIER were united in seeing the practice’s EMR system as the best place to start to enable seamless and systematic collection of patient outcome data either on-site, in the waiting room or from the web at home. Specifically the EMR could be used to collect baseline measures of quality of life, pain and other outcome measures to provide an invaluable repository of data. The primary obstacles to research within IHC centres were time and compliance of practitioners in recording data.

Other research activity within BraveNet Centres and the other clinics visited are driven by individual practitioners who champion research, or have a designated research position alongside their clinical responsibilities. Some centres were based within a University, and nearly all centres were affiliated with a University. These links strongly supported research initiatives. Ongoing issues reported for supporting research included securing funding, the difficulty in collecting costs of individual treatments, and the skills and support necessary to manage a large data repository.

Interviewees were asked to comment on the feasibility of conducting a clinical trial of an integrative health care intervention. Many interviewees agreed that research investigating individualised integrative health care for a condition or disease was an area that was very underdeveloped and needed. It moves away from “reductionist approaches to outcomes that are clinically relevant to the patient and the practitioners”.

A prominent theme that emerged was appropriate methodology. There was a sense that a complex methodology was needed to investigate the effectiveness of IHC to capture the outcomes for the “whole person” not just “disease”. Even framing the research question was considered difficult:

“So, are we looking at the global picture of evidence for patient improvement…which, it’s kind of a hard question. It’s different than do we meet the primary end point. ..Like, I think there’s plenty of trials where we don’t meet primary end point but a lot of great secondary endpoints. So, that’s one thing that evidence-based is kind of getting a backlash of…are we even asking the right question”. (Int5)

One interviewee illustrated this point with describing a patient with a head injury and post traumatic stress disorder, which may include insomnia, depression, fatigue.

“…a cluster of core aspects that have to do with emotional aspects of PTSD…co-morbidities…In the conventional system we split these up into silos. If you have a head injury you go see the neurologist, If you have a mind injury you go see the psychiatrist, if you have a body injury you go see the orthopod if it’s your leg, or the osteopath if it’s your back…and if you can’t find anything wrong with those or if there’s a fatigue issue you might go see the rheumatologist or the endocrinologist, or something like that. …Each of them then manages that one little component, usually with a medication. In some cases with a behavioural component…It’s a dependency model… So, if you then say well we want a salutogenic, rather than a pathogenic, approach… For these kinds of whole person healing modalities where you’re looking at all these co-morbidities improving, if it’s being done properly, that one can begin to frame a cost assessment around that. It’s called cost-consequence…that is something that should be used and tested”. (Int6)

Practitioners and IM leaders noted the importance of assessing the “non-specific contextual factors”, the limitations of investigating IHC in a mechanistic framework with reference to the patient-practitioner relationship:

“The problem is the ‘data’ are only available for the things that happen to be studied. Knee arthritis for acupuncture, there’s about 50 things that had actually been studied enough to say that there’s data behind them. Well, most people don’t fit into those 50 categories so you’re back to that connection for the patient with the modality. The other 50% of it we found was the practitioner. It’s absolutely a relationship between that patient and that practitioner and modality is secondary”. (Int3)

“…when assessing what made a difference in an integrative intervention, you may find that the actual therapeutic component for some might have been the interaction, and how they were presented with the different modalities, the choice they made and their expectations, the learning and framing that occurred with those particular practitioners. For example, “…say you have a practitioner that’s very good at framing things and reducing distress in a patient. That frequently correlates with improved pain outcomes”. (Int4)

When asked what outcomes measures might be appropriate in assessing an IHC intervention, the following were mentioned:

- Measure yourself medical outcome profile (MYMOP) [14];
- the one-item visual analogue Arizona Integrative Outcomes Scale (AIOS), which assesses self-rated global sense of spiritual, social, mental, emotional, and physical well-being over the past 24 hours and the past month [15]; and
- items from the Patient Reported Outcome Measures Information System (PROMIS) toolbox for the specific condition(s) [16]. PROMIS is being used to develop and validate a family of self-report measures to assess contextual factors such as attitudes towards health, the clinical environment, and patient views of the patient-provider relationship.

Other areas mentioned as lending themselves to IHC research, include assessing a constellation of cardiovascular risks, initiating an integrative healthcare intervention and then measuring across these such as lipids, and blood pressure.

## Discussion

The themes that emerged from the interviews include shared vision or philosophy [17], the changes required to organisational arrangements to deliver IHC [18], the importance of co-location, the lack of formal structures within the IHC to facilitate collaboration, the lack of guidelines or protocols [19] and the desire to incorporate a viable research component within an IHC program but limited resources to do so [20]. These, in part, reflect some observations found in other reviews of IHC primary care health clinics and team orientated initiatives within hospitals [17, 18, 20].

The extent to which an IHC “performed” in an “integrative way” varied according to the patient needs and preferences and the practitioner’s view of the perceived benefits of involving other practitioners. However, it was also influenced by the ease with which the integration could occur, that is, the organisational and clinical structures that were in place to support integration. Our report documents the variability in these supportive structures across the different IHC entities

On an organisational level, physical co-location of services and therapies was an important facilitator in integrative processes. Internal referrals were an integral part of the co-location. For patients, this means there is more opportunity for ‘seamless care’. There is the option of direct contact between care givers providing the opportunity for sharing information and knowledge, such as ‘corridor conversations’ and more formal meetings [21]. The opportunity for ‘seamless care’ was facilitated or obstructed by the different structures of the clinics. Part-time practitioners were limited in their availability for formal case conferencing and restricted to casual interactions with those practitioners that work the same days. IHC requires practitioners to expand their knowledge of other modalities, participate in case conferences, share charting, and other related case management tasks, and generally, all in addition to their usual work. This additional work is not well supported in an organisational structure where practitioners are part-time or fee-for-service as it comes at a direct financial cost.

Service delivery was integrated through shared information systems which were in place, or in the process of being implemented in all centres and hospitals. This enabled ease of communication using internal messaging or email systems. Patient intake and information was centralised through reception or web or for inpatients at admission. Typically a shared admission form was available to all practitioners. But beyond this, integrative care might be more accurately defined as “linkage” or “collaboration”. Only one centre had experimented with doing integrative patient intakes where a patient could make a special appointment and a team would attend and a treatment plan would jointly be determined. Innovation in patient intake in primary care have faced logistical problems in scheduling intakes that involve two or more practitioners, and achieving financial sustainability [22]. However, the general 15 minute consultation with a primary physician is not adequate to achieve health goals and there is evidence that augmenting consults with a health coach or similar improves outcomes [23]. There appear to be many unexplored advantages to augmenting consultations with CM practitioners and IHC approaches [24].

Different theories have been proposed to describe integrative health care. Boon (2004) proposes a continuum of seven different models ranging from parallel care to integrative care [25]. Placement along the continuum is determined by differences in philosophy, structure, process and outcomes, implicit in this theoretical framework is that it is desirable to be further along the continuum, that is “more integrative”. Within this framework, it is possible to place each IHC centre along the Boon’s continuum but this does not reflect clinical day-to-day operations where “integrative” care does occur alongside “parallel practice”. One approach that has been developed from Boon’s continuum, has been to acknowledge that more than one type of collaboration might occur at a single site, and therefore “grouping models” may be more useful [26]. Another possible theoretical framework for understanding IHC is to describe multidisciplinary care according to three tiers - *linkage*, *coordination* and *integration.* Each tier requires an increased level of organisational and clinical arrangements [27, 28]. Different tiers are appropriate for different individuals. For example, *linkage* is adequate for patients with mild to moderate care needs. Whereas for patients with complex care needs full *integration* may be required, that is, where new ‘programs’ are created from the pooling of resources. The differentiation between tiers is important as not all patients may require an integrative approach. Perhaps within the spectrum of Boon’s continuum, ‘integrative’ care needs to be contextualised as being the goal only for some patients, perhaps those with complex care needs [25]. For other patients, linkage, or coordinated or collaborative structures and processes that are based on the goals of treating the whole person may be more than appropriate [1].

The findings of our study suggest that IHC clinics struggle with the process of integrative care. There was often a mismatch between the desirable way in which an integrative health centre should operate and the economic reality. We found that beyond the shared patient intake form, structure for integrative care was loose. There was no use of protocols or guidelines within any centre. No patient monitoring mechanism beyond that which occurred within one-on-one appointments. Some joint planning may occur for a patient treatment but typically in an ad hoc way, through informal mechanisms such as corridor conversations, and short phone calls. Typically these treatment plans were “brokered” by the biomedical physician when a patient was to be seen by other practitioners within the centre. While patients could initiate direct contact with CM practitioners if desired, the tendency to biomedical dominance through control of the referral process, found in other studies, was evident [4, 29]. These barriers are similar to a study of 19 centres in Canada where key issues related to leadership, resources, philosophy and logistics [20].

Our findings on research in an IHC setting are similar to much of the literature on IHC research methodology [30, 31]. Stakeholders emphasised the need for any research undertaken to recognise that within the integrative whole person approach there is considerable potential for cost-effectiveness. Appropriate measures to capture whole person outcomes are seen as a key component of any research design by our participants.

Service delivery and the process of care within hospital inpatient services followed a more formalised structure. This is likely attributable to the clearly defined hierarchy of responsibilities for CAM practitioners and physicians. In one hospital, the scope of treatment to be provided by CAM practitioners was clearly defined, and in some cases required the sign off from the treating physician. Other studies of CM practitioners in hospital settings have identified that clear boundaries are often drawn around the ‘work’ of CM practitioners. Physicians may support the CM practitioners’ presence but maintain ‘jurisdictional’ control [1, 32, 33].

The purpose of exploring IHC centres was to provide an insight into how the efficacy of IHC may be tested in a clinical trial setting. Several trials are making progress in this emerging area [34, 35]. The findings from our review point to several factors that would influence the structure of trial: the inclusion of clear formal structures and information systems for sharing and collaborating that are not costly or burdensome, the development of a protocol to guide triage, intervention and review that is not fixed (and thereby limits clinicians and external validity) but responds to the individual needs of the patient, and set periodic reviews built into patient treatment plans. The lack of a fixed “integrative” intervention may cloud understanding of which “part” is causing the outcome, but it is likely that the whole is greater than the sum of the parts [36, 37]. Evaluating a complex health system is not without challenges but attempts are being made to do this within the context of integrative health care [38].

Our study has several limitations. The range of centres in our sample and the limited number of interviews at each centre makes it difficult to generalise our findings. However, the themes that arose in our study are not dissimilar to those that have been found in other studies [2, 20].

## Conclusions

IHC is a complex, emerging field with divergent meanings and interpretations. The IHC entities considered in this report hold the patient at the centre and fluidly use an integrative health care approach to meet that patient’s needs within resource constraints. More formalised structures and systems may facilitate this integration in the future. It is important to understand the structures and processes of IHC to provide evidence for future planning and implementation so as to meet patient needs and demands in this area. This exploration of the available models of IHC facilitates future “testing” of the efficacy of IHC delivery.
